# Mesenchymal Stem Cells-based Cell-free Therapy Targeting Neuroinflammation

**DOI:** 10.14336/AD.2023.0904

**Published:** 2024-05-07

**Authors:** Hongjie Xu, Bin Wang, Ang Li, Jing Wen, Huanxing Su, Dajiang Qin

**Affiliations:** ^1^Key Laboratory of Biological Targeting Diagnosis, Therapy and Rehabilitation of Guangdong Higher Education Institutes, The Fifth Affiliated Hospital of Guangzhou Medical University, Guangzhou, China.; ^2^Bioland Laboratory (Guangzhou Regenerative Medicine and Health Guangdong Laboratory), Guangzhou, China.; ^3^Greater Bay Area Institute of Precision Medicine (Guangzhou), Fudan University, Guangzhou, China.; ^4^State Key Laboratory of Quality Research in Chinese Medicine, Institute of Chinese Medical Sciences, University of Macau, Macao, China.; ^5^Centre for Regenerative Medicine and Health, Hong Kong Institute of Science & Innovation, Chinese Academy of Sciences; Hong Kong SAR, China

**Keywords:** neurodegenerative disease, neuroinflammation, mesenchymal stem cells, exosome

## Abstract

Emerging from several decades of extensive research, key genetic elements and biochemical mechanisms implicated in neuroinflammation have been delineated, contributing substantially to our understanding of neurodegenerative diseases (NDDs). In this minireview, we discuss data predominantly from the past three years, highlighting the pivotal roles and mechanisms of the two principal cell types implicated in neuroinflammation. The review also underscores the extended process of peripheral inflammation that predates symptomatic onset, the critical influence of neuroinflammation, and their dynamic interplay in the pathogenesis of NDDs. Confronting these complex challenges, we introduce compelling evidence supporting the use of mesenchymal stem cell-based cell-free therapy. This therapeutic strategy includes the regulation of microglia and astrocytes, modulation of peripheral nerve cell inflammation, and targeted anti-inflammatory interventions specifically designed for NDDs, while also discussing engineering and safety considerations. This innovative therapeutic approach intricately modulates the immune system across the peripheral and nervous systems, with an emphasis on achieving superior penetration and targeted delivery. The insights offered by this review have significant implications for the better understanding and management of neuroinflammation.

Neurodegenerative diseases (NDDs) are becoming increasingly prevalent worldwide. These diseases represent a heterogeneous group of neurological disorders that are predominantly age-related and progressively impair neuronal function. While these disorders can manifest in either the central nervous system (CNS) or peripheral nervous system (PNS), emerging studies suggest that pathologies in the PNS may precede CNS involvement by several years, potentially culminating in neurodegenerative disease in older individuals. Among the various hallmarks of brain aging, neuroinflammation is garnering significant attention [1].

The burgeoning field of research bridging the nervous and immune systems holds promise for understanding and potentially ameliorating a spectrum of NDDs. Historical accounts dating back to the 17th Century [2] provide initial evidence of immune responses within the nervous system. Over the ensuing decades, neuroinflammation has been recognized as a pathological hallmark of a variety of NDDs, including Alzheimer’s disease (AD), Parkinson’s disease (PD), amyotrophic lateral sclerosis (ALS), Huntington’s disease (HD), multiple sclerosis (MS), major depressive disorder (MDD) [3], and stroke [4-9]. The principal cellular regulators of the inflammatory response in brain disease consist of two primary cell types: microglia and astroglia.

## Microglia

Microglia, recognized as brain-resident macrophages, play a pivotal role in neuroinflammation. These cells represent another subtype of macrophages within the CNS, distinct from border-associated macrophages (BAMs). As the majority component of myeloid cells within the brain, microglia are integral in maintaining metabolic balance and bolstering immune defenses, mirroring the role of immune cells in the PNS [10, 11].

Microglia originate from yolk sac (YS) hematopoiesis during the embryogenic process and subsequently infiltrate the brain parenchyma [12]. Their function is modulated by the diversity of pyramidal neuron subtypes [13]. A critical aspect of microglial activity involves the elimination of toxic aggregates within the brain [14]. Through a process reliant on F-actin-dependent polymerization, microglia form an “on-demand” functional network, facilitating intercellular connections. This mechanism permits the transfer of alpha-synuclein (alpha-syn) from overburdened microglia to neighboring naive microglia, enabling efficient degradation of the alpha-syn cargo [15].

Microglia-induced neurodegeneration, termed microgliosis, has been identified in all NDDs [5, 6]. These microglial cells are noted to secrete increased levels of reactive oxygen species, proinflammatory factors, and components of the complement system [16, 17], and they undergo substantial morphological alterations leading to changes in cell structure and function [18]. Global transcriptomic analyses revealed apolipoprotein E epsilon 4 (APOE4)-driven lipid metabolic dysregulation within microglia. APOE4, a significant genetic risk factor for AD, initiates glial-specific autonomous dysregulation that may contribute to increased AD susceptibility [19]. The cerebrospinal fluid dynamics are closely intertwined with neural response within the brain. A recent investigation demonstrated that meningeal lymphatics could modulate microglia function and anti-Aβ immunotherapy [20].

Research conducted this year showed alterations in innate and adaptive immune responses in mice with tauopathy, but not in those with amyloid deposition. Interestingly, microglial or T cell deficiency impeded tau-mediated neurodegeneration [21]. This finding uncovers a tauopathy- and neurodegeneration-linked immune nexus involving activated microglia and T cell responses, proposing potential therapeutic targets for preventing neurodegeneration in AD and primary tauopathies. Moreover, dysregulated microglial ferroptosis has been implicated in PD [22], and a reduction in microglia-mediated OxPC clearance has been associated with neurodegeneration in MS [8]. Microglia in the spinal dorsal horn are activated following PNS damage, potentially instigating local neuroinflammation that may contribute to neuropathic pain processes [23].

The integrity of myelin’s structure and function is frequently compromised in aging and NDD. Given their pivotal role in maintaining myelin health during adulthood in both mice and humans, microglia present promising therapeutic targets [24]. Several studies propose potential therapeutic strategies targeting microglia. For instance, the endogenous lipid mediator Resolvin D1 (RvD1) can mitigate microglial inflammation by attenuating the activation of inflammatory signaling pathways [25]. Brilliant blue G has been shown to inhibit LPS-induced neuroinflammation in microglia by modulating MAPKs and NF-kappaB signaling pathways [26]. Additionally, encouraging the polarization of microglia phenotypes toward M2 with curcumin treatment can decrease neuroinflammatory responses, possibly inhibiting the TLR4/MyD88/NF-kappaB signaling pathway following subarachnoid hemorrhage [27].

## Astrocytes

Microglia intimately interact with and stimulate astrocytes, a process crucial for maintaining immune homeostasis. Microglia play a role in facilitating astrocyte proliferation and the formation of astrocytic scars, and microglial depletion can augment inflammatory responses, primarily by reducing STAT3 phosphorylation in astrocytes following spinal cord injury [28]. The secretion of fragmented mitochondria from microglia can activate astrocytic responses and instigate neuroinflammation [29]. Reactive astrocytes, in turn, can amplify microglial inflammatory responses via the fibronectin/beta1 integrin pathway within the glial scar following spinal cord injury [30]. The intricate crosstalk between astrocytes and microglia is regulated by several factors, with IL-3 identified as a significant cytokine, and thus a potential therapeutic target, in AD [31]. In fact, a lymphocyte-microglia-astrocyte axis has been identified in chronic active MS [32].

Astrocyte activation plays a fundamental role in neurodegenerative diseases [33-35]. Similar to microglia, astrocytes express DNA sensors in both viral infection and sterile inflammatory responses, which include the PYHIN family members AIM2, IFI16, and p204, as well as the enzyme, cGAS, exacerbating existing neuroinflammation [36]. Some calcium channels can activate astrocytes via Connexin-43 mediation in vincristine-induced neuropathic pain [37]. A marked increase in all inflammatory markers (interferon-gamma, IL-12p70, IL-1beta, IL-2, IL-4, IL-6, TNF-alpha, and IL-17A), with the exception of IL-10, was identified in both astrocyte-derived extracellular vesicles and the serum of NDD patients [3]. The accumulation of lipid droplets (LDs) leads to mitochondrial damage and induces the secretion of inflammatory factors in astrocytes, a mechanism implicated in NDD pathogenesis [38]. Reactive astrocytes also propagate inflammation in a blood-brain barrier (BBB) model, partially through a TNF-STAT3 signaling pathway axis [39]. DNA damage is a significant contributor to neurotoxic inflammation in astrocytes in Aicardi-Goutières syndrome [40]. Recent studies suggest that astrocyte reactivity is an early marker linking Aβ with initial tau pathology, potentially serving as an indicator of early-stage AD and a criterion for clinical trial recruitment [41]. Through RNA-sequencing data analysis, novel genes related to inflammation have been identified in astrocytes, oligodendrocytes, and microglia in MS [42]. Furthermore, translatome data indicates that microglia and astrocytes have distinct roles in inflammation during the hyperacute and acute phases following stroke [43]. Collectively, these findings provide evidence linking glial activation to the progression of NDD.

Astrocytes represent a direct target for anti-inflammatory interventions. The release of APOE isoforms from astrocytes and microglia is a well-established therapeutic intervention against inflammation [44]. The APOE-TAGLN3-NF-kappaB signaling pathway mediates neuroinflammation within astrocytes. Moreover, TAGLN3 modulation of neuroinflammation holds promise as a biomarker for AD [45]. The activity of MAFG within astrocytes instigates CNS inflammation, offering a further target for interventions against MS [46, 47]. Hydrogen sulfide’s neuroprotective impact on astrocytes opens up avenues for developing novel therapeutic strategies for NDDs [48]. Ferrostatin-1 has been shown to ameliorate ferroptosis in astrocytes, consequently inhibiting angiotensin II (Ang II)-induced inflammation [49]. miR-128-3p has been found to mitigate penicillin-induced apoptosis and restrict inflammation in astrocytes implicated in epilepsy by modulating the MAPK6 pathway [50]. Inhibiting P38MAPK has been shown to attenuate the response of astrocytes, resulting in decreased inflammation in aged astrocytes [51]. Similarly, miR-499a targeting PTEN in astrocytes has demonstrated comparable anti-inflammatory effects in the context of ischemic stroke [52].

## Inflammation of the peripheral system

Peripheral inflammation significantly influences the neural system and contributes to the development of NDDs. Acute inflammation in the PNS has been linked to enhanced neuroinflammation in AD, as substantiated by research showing that peripheral IL-1beta can stimulate amplified responses in primed astrocytes, thereby leading to neuronal network dysfunction [53]. A recent review has approached blood-to-brain communication from a systems physiology perspective, highlighting the potential role of blood-derived signals as powerful contributors to NDDs [54].

In addition to the aforementioned microglial cells and astrocytes, the balance of neuroinflammation can also be modulated by other cells, such as PNS macrophages and a variety of immune cells that reside within the brain’s microenvironments. Increasing evidence has underscored their roles as agents of brain aging and cognitive decline. For instance, recent research has provided evidence that age-dependent structural dysregulation of myelin can lead to Aβ plaque formation [55]. Another study has proposed that maintaining oligodendrocyte health and myelin integrity may represent a promising approach to slowing the progression of AD [55]. A new theoretical framework, referred to as the “neuro-glia-vascular unit” (NGVU), has emerged recently. This concept seeks to encapsulate the intricate interactions among vascular cells, glial cells, and neurons [56].

## Anti-inflammatory effects of MSCs and exosomes

Stem cells (SCs) play an integral role in maintaining tissue homeostasis, the imbalance of which, with advancing age, can contribute to inflammatory degenerative niche and disease manifestation [57-59]. Known for their dual inflammatory modulation capabilities, MSCs interact with a broad spectrum of immune cells, including astrocytes and microglia. Recent studies have further corroborated the anti-inflammatory potential of MSC-based interventions across a range of physiological systems, including integumentary, pulmonary, ocular, hepatic, skeletal, metabolic, and neural systems [60-69].

The reciprocal communication between MSCs and the immune system is facilitated by extracellular vesicles, particularly across extended distances [65-67, 70]. Of particular interest are small extracellular vesicles (sEVs) derived from human umbilical cord mesenchymal stem cells (hUC-MSCs). These sEVs have demonstrated effectiveness in modulating the inflammatory response and promoting neuro-regenerative processes in SCI. Furthermore, these vesicles have been observed to induce polarization from the proinflammatory M1 macrophage phenotype to the anti-inflammatory M2 subtype, both in vitro and within lesion sites in rat models of compressive and contusive SCI [67, 71].

Exosomes derived from MSCs, when primed using physical and chemical methodologies such as hypoxia, traditional Chinese medicine, gene editing, and three-dimensional culture, exhibit enhanced functional characteristics [65-67, 70-72].

## Regulation of MSCs on microglia and astrocyte

Evidence demonstrates that MSCs, subject to physical, chemical, and biological stimuli, can differentiate into neural cells [73-77]. BMSCs have been shown to differentiate into neurons, astrocytes, and oligodendrocytes, thereby attenuating inflammation and demyelination in EAE mouse models [78, 79].

MSCs have also been reported to regulate microglial pyroptosis following intracerebral hemorrhage in rodent models, primarily by targeting C1q/tumor necrosis factor-related protein 3 [80, 81]. A preclinical investigation underscored the pivotal role of controlled, localized transplantation of MSCs, which secrete the anti-inflammatory cytokine IL13, thus modulating microglial and macrophage responses following ischemic stroke [82]. Notably, MSCs have been found to influence the transcriptional conversion of microglia/macrophages, promoting the acquisition of the M2 subtype associated with tissue repair and growth stimulation, in both in vitro and in vivo models of traumatic brain injury [83].

Additionally, MSCs can mediate astrocytic responses. They have been found to reduce inflammation and demyelination by downregulating A1 neurotoxic reactive astrocytes in the chronic cuprizone demyelination model while concurrently enhancing the secretion of BDNF and TGF-beta as trophic factors [84].

**Figure 1.**
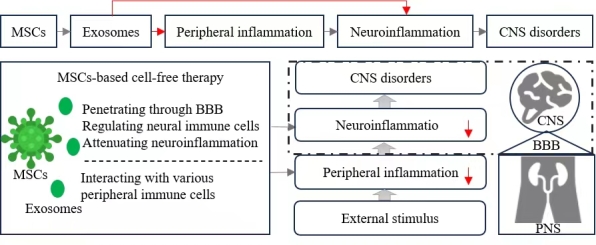
**Mesenchymal stem cells (MSCs)-based therapy targeting neuroinflammation**. MSCs interact with various types of immune cells in the peripheral system and release exosomes that modulate both peripheral and neural inflammation. These exosomes possess enhanced penetrative abilities and safety profiles.

## MSCs regulate peripheral immune cells

MSCs interact with various types of immune cells in the peripheral system, including T cells, B cells, and macrophages. The regulation of interactions between macrophages and MSCs plays a pivotal role in a broad range of physiological functions [85-90]. Aging introduces significant alterations to the composition and functionality of peripheral immune cells, leading to increased local and systemic inflammation. Evidence derived from parabiosis experiments and bone marrow reconstitution trafficking studies suggests that aging facilitates the infiltration of circulating immune cells into the brain, consequentially impeding cognitive and regenerative processes [91, 92].

## Superior penetration and targeting of anti-neuroinflammatory exosomes from MSCs

MSCs have shown remarkable migratory capabilities to the brain, even without intracranial transplantation. In studies involving intranasal delivery of MSCs to transgenic PD and AD mouse models, migration to various brain regions including the olfactory bulb, cortex, amygdala, striatum, hippocampus, cerebellum, and brainstem was evident within seven days [93]. Importantly, Interleukin-1beta-preconditioned MSCs have demonstrated an ability to attenuate inflammation in hippocampal astrocytes, mediated via exosome-induced Nrf-2 signaling [94]. Additionally, BMSCs treated under hypoxic conditions have displayed enhanced anti-inflammatory properties, which are beneficial in mitigating OGD/R-induced injury, partially attributable to exosome-facilitated microglia polarization [95]. These findings not only enhance our understanding of the pathophysiological mechanisms underlying NDDs but also provide a promising avenue for exploring novel therapeutic interventions for inflammatory brain diseases (Fig. 1).

## MSCs’ exosome in treating AD

The therapeutic application of MSC-derived exosomes in mitigating AD-related neuropathology and alleviating neuroinflammation has been established through studies conducted in animal models. These exosomes display neuroprotective properties and mitigate Aβ-induced neurotoxicity [96, 97]. They further facilitate neurogenesis and ameliorate neuroinflammation in AD mouse models. Remarkably, MSC-derived exosomes procured from three-dimensional cultures have demonstrated the capability to eliminate Aβ plaque and neuropathology in 5 × FAD mice [98]. The modulatory effects of MSC-derived exosomes on AD-related neuropathology are predominantly attributed to their molecular cargo. These exosomes carry anti-inflammatory molecules such as PGE2, TGFβ, and IL-10, which suppress inflammation by regulating the activities of astrocytes and microglia [99-103]. Exosomes overexpressing miR-199a-5p, derived from BMSC, have been found to promote NSC proliferation via the GSK-3β/beta-catenin signaling pathway [104]. Furthermore, H19 carried in BMSC-derived exosomes has been observed to modulate LPS-stimulated microglial M1/M2 polarization [105]. Recent findings have corroborated the feasibility of delivering MSC-sEV to the injured neonatal brain, which confers protection partially through their modulatory effects on microglial cells [106]. Moreover, MSC-EVs have been shown to regulate astrocytic responses and mitigate neurotoxicity through their anti-inflammatory properties and microRNA cargo, thereby demonstrating therapeutic potential in ALS [107].

## Engineering of exosomes in AD treatment

Despite the potential of exosome therapy in clinical applications, the technique continues to face challenges in the form of separation methodologies, batch-to-batch variability, and limited scale of production. Engineering approaches may serve to augment the therapeutic potential of exosomes in AD therapy. These innovative strategies encompass parental cell preconditioning to amplify specific functionalities, incorporation of therapeutic cargo, and surface modifications. MSCs preconditioned by hypoxia are observed to exhibit heightened anti-inflammatory attributes. The hypoxia-induced microRNA, miR-21, is also significantly expressed in exosomes derived from hypoxia-preconditioned MSCs [108]. Such exosomes have shown promising potential in enhancing cognitive function in APP mice models [109].

Furthermore, overexpression of select neuroprotective genes in MSCs can lead to an enriched presence of these genes in the derived exosomes. Notably, exosomes encapsulating miR-22 and SHP2 have demonstrated therapeutic effects in Alzheimer’s disease by suppressing specific genes, promoting neuronal regeneration, curtailing inflammation, and augmenting memory abilities in mouse models [110, 111].

Additionally, peptides originating from various sources have been utilized to facilitate the entry of nanoparticles into the brain. This strategy could also be employed to enhance the ability of exosomes to target the brain [112]. Current research is investigating the use of cell-penetrating peptides like RVG to enable blood-brain barrier penetration and entry into neuronal cells via specific receptors like acetylcholine or GABA receptors, present in microvascular endothelial cells and neurons [113].

## Safety of MSC treatments on brain disease

Pluripotent stem cells (PSC) present vast potential for cell therapies aimed at numerous diseases, including NDDs [114]. Nevertheless, PSC therapy faces challenges such as tumorigenicity, immunogenicity, and heterogeneity [114, 115]. Instead, MSCs are increasingly favored due to their superior safety record. As of June 2023, over 1,100 projects have been registered on clinicaltrials.gov, with 111 studies focusing on MSCs in brain diseases. By May 31, 2023, 43 clinical trials conducted by 29 Chinese companies received provisional approval from the NMPA (www.nmpa.gov.cn/) for MSC-related medications. Of these, four trials are concentrated on brain diseases and three on strokes. Despite advancements in animal models and clinical trials, the application of stem cell therapy is limited. Further work is required to address issues in clinical trials, including smaller sample sizes, the absence of controls, brief follow-up periods, and single-center focus [116-118].

However, a keyword search for “exosome” and “brain” yielded only three clinical studies using exosomes for brain disorders. MSC-derived exosomes have been employed to ameliorate disabilities in acute ischemic stroke patients, with phase I and II clinical trials currently underway. The potential use of acupuncture-induced exosomes for treating post-stroke dementia has been explored, albeit the trial phase is listed as “Not Applicable.” Furthermore, exosomes from allogenic adipose MSCs have been tested for safety and efficacy in AD patients and are currently in phase I and II clinical trials. The comprehensive feasibility and safety of exosome therapy have not been definitively established through clinical studies, although a large study is expected in the near future.

To enhance the efficacy of MSCs, as well as extracellular vesicles derived from MSCs, they are typically primed prior to transplantation. This process employs various strategies, including small molecule compounds, growth factors, biomaterials, and physicochemical stimulation [68, 69, 119-124]. These techniques have demonstrated increased effectiveness against inflammatory diseases [125], implying that the therapeutic functionality of MSCs largely depends on their paracrine activity, rather than cell differentiation.

## Future perspective

MSC-EVs contain a complex and currently ill-defined array of beneficial molecules. To optimize therapeutic efficacy, it is important to characterize these components thoroughly, excising any undesirable constituents. Moreover, the cellular source and culture conditions of the EVs need to be taken into account as the origins and physiological state of parent cells can influence the composition of EVs. The capacity for cell yield, survival, and differentiation varies across different MSC sources, implying a potential impact on EV composition and efficacy. Thus, further research is needed to pinpoint the exact components of MSC-EVs contributing to AD therapy. At present, a direct comparison of EV components derived from disparate MSCs is nonexistent, leaving the differences in their therapeutic efficacy unclear. The application of multiomics techniques promises a comprehensive elucidation of EV composition and a deeper understanding of their mechanism of action in AD treatment. MSC-based therapy is garnering considerable interest; however, certain complexities need addressing. With a paucity of clinical efficacy data and mechanistic research, substantial gaps in knowledge persist. Given their nature as biological therapeutics, the manufacturing and quality control of cell products pose more challenges than their small molecule counterparts. Fortunately, a majority of preclinical studies have not indicated significant disparities in in vitro or in vivo efficacy between freshly cultured and cryopreserved MSCs [126]. Furthermore, the route of administration for brain disorders necessitates a careful evaluation of BBB considerations. The potential and advantages of cell-free therapy are increasingly recognized and could offer a compelling alternative to other strategies [124, 127, 128].

## Conclusion

Chronic neurodegenerative diseases associated with aging represent a complex and diverse group of neurological disorders that evolve over extended periods. Decades of rigorous research have significantly enhanced our understanding of their pathophysiology. Recent reviews have summarized advances in the dynamics of the extracellular matrix and the regulation of intracellular transcription factors [129, 130]. A growing body of research has shifted focus toward the role of neuroinflammation, providing novel insights that could potentially impede or inhibit the progression of such age-related brain disorders [131, 132]. MSCs, particularly the MSCs-based cell-free therapy, have emerged as a potent therapeutic strategy. The dual functions of exosomes in neurodegenerative conditions have been discussed in a recently published review [133]. Both in our own unpublished research and in the work of others, the significance of enhanced stem cells and their exosomes is under active investigation [134]. Their notable anti-inflammatory properties warrant closer scrutiny and comprehensive study, underscoring their potential in mitigating the impact of age-related neurodegenerative diseases.
